# Functional Assessment Beyond Type A Tympanograms: Pressure-Swallow Testing for Chronic Eustachian Tube Dysfunction in a Taiwanese Cohort

**DOI:** 10.3390/diagnostics16172738

**Published:** 2026-08-26

**Authors:** Chen-Yi Lu, Jing-Jie Wang

**Affiliations:** 1 Department of Otolaryngology, Taichung Veterans General Hospital, Taichung 407219, Taiwan; chenyilu0120@vghtc.gov.tw; 2 School of Medicine, National Yang Ming Chiao Tung University, Taipei 112304, Taiwan

**Keywords:** Eustachian tube dysfunction, pressure-swallow test, tympanometry, impedance audiometry, Eustachian Tube Dysfunction Questionnaire-7 (ETDQ-7), maximal peak pressure difference (MPD)

## Abstract

**Background:** Eustachian tube dysfunction (ETD) is associated with several common otologic conditions but lacks standardized diagnostic thresholds in Taiwan. This study aimed to evaluate the diagnostic performance of the GSI TympStar Pro pressure-swallow test and to identify a ROC-derived maximal peak pressure difference (MPD) cutoff for distinguishing clinically diagnosed obstructive Eustachian tube dysfunction (oETD) from healthy controls. **Methods:** A total of 152 subjects were enrolled, including 100 healthy controls and 52 patients with clinically diagnosed oETD, confirmed by otolaryngologist assessment and supported by ETDQ-7 symptom scoring. Tympanometry and tympanometry-based Eustachian tube function testing using the GSI TympStar Pro ETF–Intact pressure-swallow module, a modified three-tympanogram pressure-swallow protocol, were performed. Group comparisons were conducted using Mann–Whitney U and Chi-square tests. Diagnostic performance was assessed using receiver operating characteristic (ROC) curve analysis. **Results:** The median MPD was 11 daPa (IQR 6–17) in the control group, compared to 0 daPa (IQR 0–2) in the ETD group (*p* < 0.001). ROC analysis demonstrated that a cutoff value of ≤4 daPa yielded a sensitivity of 100.0% and specificity of 91.0% in the per-person analysis, and a sensitivity of 97.5% and specificity of 95.5% in the per-ear analysis. In the Type A-only per-ear analysis, MPD retained discriminatory performance, with an AUC of 0.978 (95% CI, 0.961–0.994). **Conclusions:** When interpreted in conjunction with ETDQ-7 symptom assessment and routine clinical evaluation, pressure-swallow testing may provide complementary objective information for the functional assessment of patients with suspected obstructive Eustachian tube dysfunction, particularly when resting tympanometry is unremarkable. In this single-center Taiwanese cohort, lower MPD values may serve as an adjunctive indicator of impaired Eustachian tube pressure equalization, and the ROC-derived cutoff of ≤4 daPa showed discriminatory value for differentiating symptomatic obstructive ETD from healthy controls. This cutoff should be interpreted as an exploratory, protocol-specific threshold and requires prospective validation in larger multicenter populations before broader clinical application.

## 1. Introduction

The Eustachian tube (ET) is a critical anatomical structure responsible for maintaining pressure homeostasis, ventilation, and mucociliary clearance in the middle ear [1]. Through periodic opening during swallowing and other physiologic activities, the ET enables equilibration of middle ear pressure with ambient atmospheric pressure, thereby preserving normal tympanic membrane mobility and efficient sound transmission [2]. Dysfunction of this structure, known as Eustachian Tube Dysfunction (ETD), is a common clinical condition characterized by a constellation of subjective symptoms, including aural fullness, otalgia, pressure, and crackling sounds. Patients may additionally report muffled hearing, discomfort during ambient pressure changes, or impaired pressure equalization, reflecting disruption of normal middle ear ventilation and pressure regulation [3,4]. Beyond patient discomfort, ETD is a significant contributing factor to chronic middle-ear pathologies such as otitis media with effusion (OME), tympanic membrane retraction, and chronic otitis media [5], frequently leading to conductive hearing loss or increased listening effort [6,7,8].

Despite its clinical relevance, the diagnosis of ETD remains challenging because no universally accepted gold-standard objective test currently exists, and available assessment tools often demonstrate variable diagnostic performance across different clinical presentations. Furthermore, ETD represents a heterogeneous spectrum of disorders involving abnormalities in pressure regulation, ventilation, and tubal opening dynamics, making accurate functional characterization difficult using any single-assessment modality. Current diagnostic approaches therefore rely on a combination of clinical history, symptom evaluation, otoscopic findings, and various objective tests, including tympanometry, tubomanometry, sonotubometry, and pressure-based functional assessments. However, each method evaluates only selected aspects of Eustachian tube physiology and has inherent limitations in sensitivity, specificity, reproducibility, or applicability across different disease states. Consequently, objective assessment of ET function remains an unmet clinical need, particularly in patients with persistent symptoms but inconclusive conventional findings [9,10,11].

In Taiwan, the Eustachian Tube Function module of the GSI TympStar Pro system is widely used in tertiary referral centers for clinical assessment of ET function. This module utilizes a device-implemented modified three-tympanogram pressure-swallow procedure based on the inflation–deflation principle of Eustachian tube function testing to evaluate middle ear pressure equilibration during swallowing and provide dynamic functional information beyond conventional resting tympanometry [12].

The primary objective parameter derived from this test is maximal peak pressure difference (MPD), which reflects the ability of the Eustachian tube to equilibrate middle ear pressure during swallowing and has been widely used as an indicator of ET function [13,14]. In general, higher MPD values indicate more effective pressure equalization, whereas lower values may suggest impaired tubal opening or reduced ventilatory capacity. Previous studies have proposed normative MPD cutoff values ranging from approximately 10 to 15 daPa to distinguish normal from impaired ET function, although reported thresholds vary across studies, likely due to differences in study populations, diagnostic criteria, device settings, and testing protocols [12,15,16,17]. For example, Bae et al. identified 13 daPa as the optimal cutoff value in their cohort [15]. These findings suggest that interpretation of MPD may require consideration of population-specific characteristics. Notably, observations from Taiwanese clinical practice have indicated that lower MPD values may still be compatible with normal ET function, raising questions regarding the applicability of currently available reference values across different populations [12]. This discrepancy underscores the need to evaluate population-specific MPD cutoff values and to clarify their clinical interpretation within specific testing protocols.

Therefore, this study aimed to evaluate the diagnostic performance of the pressure-swallow test implemented in the GSI TympStar Pro ETF–Intact module and to identify a ROC-derived MPD cutoff for distinguishing clinically diagnosed symptomatic oETD from healthy controls in a Taiwanese cohort. Specifically, we sought to determine the ability of MPD to discriminate patients with clinically diagnosed oETD from healthy individuals and to examine whether previously proposed reference thresholds are applicable to this population. By comparing objective impedance-based measurements between patients with oETD and healthy controls, this study aimed to provide preliminary evidence to support the clinical interpretation of pressure-swallow test results in routine practice. Rather than establishing a universal diagnostic standard, the proposed cutoff should be interpreted as an exploratory, cohort-specific, and protocol-specific discriminatory threshold that may help identify impaired Eustachian tube pressure equalization when used in conjunction with symptom assessment and routine clinical evaluation. In addition, as objective assessment of ET function has become increasingly relevant for patient selection and outcome evaluation in Balloon Dilation Eustachian Tuboplasty (BDET), population-specific normative values may contribute to more appropriate identification of candidates for interventional treatment and optimize clinical decision-making [3,18,19].

## 2. Materials and Methods

### 2.1. Study Design and Ethical Approval

This prospective case–control study was conducted from 12 March 2024 to 29 October 2024. The study protocol was approved by the Institutional Review Board of Taichung Veterans General Hospital (IRB No. CE23519C), and all participants provided written informed consent.

### 2.2. Participants

A total of 152 subjects were enrolled from the Audiology Evaluation Center and Otolaryngology outpatient clinics at a public tertiary referral medical center in central Taiwan and allocated into two groups: healthy controls (*n* = 100) and patients with ETD (*n* = 52).

For the per-person analysis, the ETD group included 52 patients. For the per-ear analysis, the ETD group included 79 affected ears from these 52 patients because some patients had bilateral involvement. Healthy controls were recruited to achieve an approximately balanced sex distribution and broad age representation (21–79 years), thereby improving the representativeness of the study cohort.

### 2.3. Procedures

All participants completed the traditional Chinese version of the seven-item Eustachian Tube Dysfunction Questionnaire (ETDQ-7) [20] to provide a standardized subjective assessment of ETD symptoms. Subsequently, all participants underwent otoscopic examination and impedance testing using the GSI TympStar Pro system (Grason-Stadler, Eden Prairie, MN, USA). The system was used to perform both conventional resting tympanometry for static assessment of middle ear status and pressure-swallow testing for dynamic assessment of Eustachian tube function.

### 2.4. Eustachian Tube Dysfunction Questionnaire (ETDQ-7)

Subjective assessment of ETD was performed using the ETDQ-7, a validated disease-specific instrument developed to quantify symptom severity in adult patients with ETD [21]. The questionnaire evaluates seven common ETD-related symptoms, including ear pressure, otalgia, aural fullness, symptom aggravation during upper respiratory tract conditions, crackling or popping sensations, tinnitus, and muffled hearing.

Each item is scored using a 7-point Likert scale ranging from 1 (no problem) to 7 (severe problem), based on symptom severity over the preceding month, yielding a total score ranging from 7 to 49, with higher scores indicating greater symptom burden.

For this study, the Mandarin version of the ETDQ-7 validated by Lin et al. was adopted to minimize linguistic and cultural bias. Based on previous validation studies demonstrating diagnostic utility of the questionnaire in identifying ETD, a total ETDQ-7 score of ≥14 was used to define the ETD group [20].

### 2.5. Tympanometry

Conventional tympanometry was performed using the GSI TympStar Pro system (Grason-Stadler, Eden Prairie, MN, USA) as an objective assessment of middle ear status. Tympanometry evaluates middle ear pressure and tympanic membrane mobility by measuring acoustic admittance across varying ear canal pressures and provides a static assessment of middle ear function. Tympanometric findings were classified according to standard tympanogram patterns as Type A, B, or C [22].

### 2.6. Pressure-Swallow Test

The pressure-swallow test is a dynamic method for evaluating Eustachian tube (ET) function. In this study, dynamic assessment of ET function was performed using the ETF–Intact pressure-swallow module implemented in the GSI TympStar Pro system. This device-implemented protocol is a modified three-tympanogram pressure-swallow pro-cedure based on the inflation–deflation principle underlying Bluestone’s Eustachian tube function testing framework, and should be distinguished from the full classical Bluestone nine-step test [12].

The test was performed using a fixed automated sequence. First, a baseline tym-panogram was obtained at ambient ear-canal pressure, corresponding to 0 daPa. After completion of the baseline measurement, the device applied positive ear-canal pressure of +400 daPa, and the participant was instructed to perform one dry swallow. The device then proceeded directly toward the negative-pressure condition without a separate re-turn-to-ambient-pressure step. Negative ear-canal pressure of −400 daPa was subse-quently applied, and the participant was instructed to perform a second dry swallow. Thus, each ear-level test consisted of one baseline measurement followed by positive- and negative-pressure swallow conditions using two dry swallows in total. All tympanograms were recorded using the same sweep direction to minimize hysteresis effects, according to the manufacturer’s recommendations.

Each ear was tested once when a technically valid recording was obtained. The test was repeated only when the recording was technically invalid, such as when ear-canal pressure could not be maintained because of probe-seal instability or when the participant was unable to complete the instructed dry-swallow maneuver. Recordings that remained technically invalid after repeat testing were excluded before analysis. No fixed rest interval was applied between repeated runs or between ears. Before testing the contralateral ear, the examiner confirmed that the participant was able to complete two additional dry swallows. If the participant had difficulty performing additional dry swallows because of insufficient saliva, the participant was allowed to sip water before the next ear was tested; however, the swallows performed during the pressure-swallow test itself were dry swallows.

Pressure loading creates a pressure gradient across the tympanic membrane. When the ET opens during swallowing, middle-ear pressure is partially equalized, resulting in a measurable shift in tympanometric peak pressure relative to the baseline measurement. The maximal peak pressure difference (MPD) was defined as the largest absolute shift in tympanometric peak pressure observed after positive- or negative-pressure loading relative to the baseline tympanogram. Higher MPD values indicate greater middle-ear pressure equalization during swallowing, whereas lower MPD values suggest reduced ET opening or impaired pressure equilibration.

For ears with measurable tympanometric peaks, MPD values were generated au-tomatically by the GSI TympStar Pro system using standardized testing procedures. In contrast, ears with Type B tympanograms were considered clinically valid recordings but had no measurable tympanometric peak. For these ears, MPD could not be directly calculated from peak pressure displacement. Therefore, they were recorded as having no measurable pressure shift and were assigned an MPD value of 0 daPa for analysis. This approach was used to retain ears without a measurable peak in the ROC dataset and to avoid excluding non-peaked or more severely affected ears from the clinical spectrum of ETD.

The pressure-swallow test was not performed under formally blinded conditions. However, because MPD was an instrument-derived quantitative parameter based on device-recorded tympanometric peak pressure changes rather than a subjective examiner-rated measurement, observer-dependent measurement bias was minimized.

### 2.7. Inclusion Criteria for the Healthy Control Group

(1) Absence of any otologic or sinonasal disease, (2) an ETDQ-7 score < 14 [21], and (3) a normal Type A tympanogram.

### 2.8. Inclusion Criteria for the ETD Group

(1) A physician-confirmed diagnosis of chronic ETD by an otolaryngologist based on persistent symptoms (e.g., aural fullness, popping, or pressure) lasting more than 3 months, and (2) an ETDQ-7 score ≥14 [21].

The ETDQ-7 score was used as a symptom-severity requirement for group allocation but not as the sole basis for diagnosing ETD. Final diagnosis of ETD was determined by otolaryngologist clinical assessment integrating symptoms, otoscopic findings, and tympanometric evaluation. Tympanometric findings (Type A, B, or C) were recorded but were not used as inclusion or exclusion criteria. Because an ETDQ-7 score of ≥14 was used as a symptom-severity requirement for ETD group allocation, comparisons of ETDQ-7 scores between groups were interpreted as confirmation of symptom burden and group validity rather than as independent evidence of diagnostic performance.

For the ear-level analysis, ears were classified as ETD ears when they corresponded to the symptomatic side identified during otolaryngologist assessment in patients meeting the predefined patient-level ETD criteria. In patients with unilateral symptoms, only the symptomatic ear was included as an ETD ear. In patients with bilateral symptoms, both ears were included as ETD ears. The contralateral asymptomatic ear in patients with unilateral symptoms was not included in the ETD ear-level dataset. Tympanogram type was recorded descriptively and was not used as an ear-level inclusion criterion.

Exclusion criteria for all participants included: (1) age <18 years, (2) perforated tympanic membrane, (3) presence of tympanostomy tubes, (4) acute otitis media, (5) recent ear surgery (within 3 months), or (6) inability to perform the test maneuvers.

### 2.9. Statistical Analysis

All statistical analyses were performed using IBM SPSS Statistics for Windows, version 22.0 (IBM Corp., Armonk, NY, USA). Descriptive statistics were generated for all study variables. Given the non-normal distribution of continuous variables, group comparisons for age, ETDQ-7 scores, and maximal peak pressure difference (MPD) were conducted using the Mann–Whitney U test [23], while categorical variables (sex) were compared using the Chi-square test [24].

For the per-person analysis, the ear demonstrating the lowest MPD value, representing the worst-performing ear, was selected to represent each participant. This approach was chosen because ETD may be unilateral or asymmetric, and averaging both ears could dilute clinically relevant dysfunction in the more affected ear. Therefore, the lowest MPD value was considered the most clinically relevant patient-level indicator of impaired Eustachian tube pressure equalization.

For the per-ear analysis, all eligible ears from both groups were included in the evaluation. Because some participants contributed both ears to the ear-level dataset, the per-ear analysis was considered supplementary and exploratory.

Because the ETD group was significantly older than the control group, an exploratory age-stratified sensitivity analysis was performed to evaluate whether the difference in MPD between groups persisted within comparable age strata. Participants were stratified into three age groups: 20–30 years, 31–46 years, and >46 years. Within each age stratum, age, ETDQ-7 score, and MPD were compared between the control and ETD groups using the Mann–Whitney U test.

Receiver operating characteristic (ROC) curve analysis [25] was performed to evaluate the diagnostic performance of MPD and to determine the optimal cutoff value using the Youden Index [26]. The per-person ROC analysis was considered the primary analysis, whereas the per-ear ROC analysis was considered supplementary and exploratory. To further evaluate whether the discriminatory performance of MPD was independent of abnormalities already detectable on conventional tympanometry, an additional Type A-only per-ear ROC analysis was performed using only ears with Type A tympanograms. Statistical significance was defined as a two-sided *p*-value < 0.05.

## 3. Results

### 3.1. Participant Demographics and Baseline Characteristics

Participant demographics and baseline characteristics are summarized in Table 1. A total of 152 participants were included in the study, comprising 100 healthy controls and 52 patients with ETD for the per-person analysis. Compared with controls, participants in the ETD group were significantly older (median [IQR], 54.5 [38.5–66.0] vs. 32.0 [27.0–40.3] years; mean ± SD, 52.1 ± 16.5 vs. 36.0 ± 12.9 years; *p* < 0.001), whereas sex distribution was comparable between groups (*p* = 0.305).

To explore the potential influence of age imbalance between groups, an age-stratified sensitivity analysis was performed (Table S1). Within each age stratum, age did not differ significantly between the control and ETD groups. However, MPD remained significantly lower in the ETD group than in the control group across all age strata: 20–30 years (1.5 [IQR, 0.0–3.8] vs. 13.0 [IQR, 9.0–20.0] daPa, *p* < 0.001), 31–46 years (0.0 [IQR, 0.0–2.3] vs. 8.0 [IQR, 5.0–14.0] daPa, *p* < 0.001), and >46 years (0.0 [IQR, 0.0–2.0] vs. 11.5 [IQR, 5.3–22.5] daPa, *p* < 0.001). These findings suggest that the observed MPD was not solely explained by the overall age imbalance between groups.

For the per-ear analysis, 200 ears from the control group and 79 ears that fulfilled the ETD criteria were included. Baseline tympanometric findings differed significantly between groups (*p* < 0.001). All ears in the control group demonstrated Type A tympanograms (100.0%), whereas the ETD group exhibited a broader distribution of tympanometric patterns, including Type A (43.0%), Type B (38.0%), and Type C (19.0%).

### 3.2. Symptom Characteristics and Objective Pressure-Swallow Test Findings in the Control and ETD Groups

**Figure 1:**
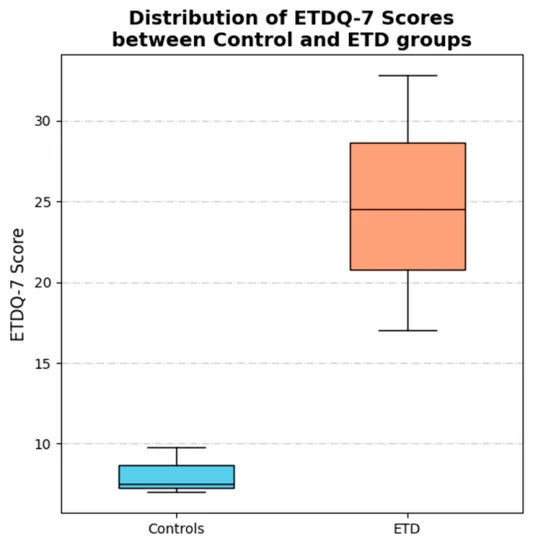
Box plot comparing Eustachian Tube Dysfunction Questionnaire-7 (ETDQ-7) scores between the control group (*n* = 100) and the ETD group (*n* = 52). The central horizontal lines represent the medians, and the boxes indicate the interquartile ranges (IQRs). The ETD group exhibited a significantly higher median ETDQ-7 score than the control group (24.5 [IQR, 17–32.8] vs. 7.5 [IQR, 7–9.8], *p* < 0.001). ETD: Eustachian Tube Dysfunction; ETDQ-7: Eustachian Tube Dysfunction Questionnaire-7.

Baseline symptom severity and objective pressure-swallow test results are summarized in Table 2. Subjective symptom burden, as measured by the ETDQ-7, differed significantly between groups. The ETD group demonstrated a markedly higher median ETDQ-7 score than the control group (24.5 [IQR, 17–32.8] vs. 7.5 [IQR, 7–9.8], *p* < 0.001), indicating substantially greater symptom burden among participants with clinically diagnosed ETD. As illustrated in Figure 1, ETDQ-7 scores were higher in the ETD group, confirming the expected difference in symptom burden between the predefined groups. Because ETDQ-7 score was part of the group allocation criteria, this comparison was presented as descriptive baseline symptom information and was not interpreted as independent diagnostic evidence.

**Figure 2:**
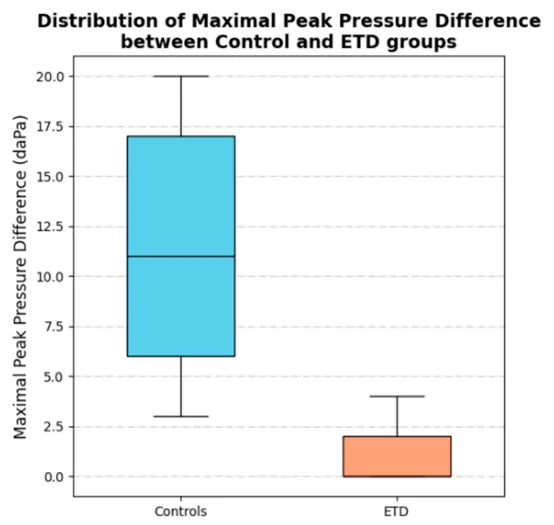
Distribution of Maximal Peak Pressure Difference. Box plot showing the distribution of maximal peak pressure difference (MPD) values between the control and ETD groups in the per-person analysis, using the worst-performing ear for each participant. MPD values were significantly higher in the control group than in the ETD group (11 daPa [IQR, 6–17] vs. 0 daPa [IQR, 0–2], *p* < 0.001). The horizontal line indicates the median; the box represents the interquartile range (IQR).

For objective functional assessment, per-person analysis was performed using the worst-performing ear, defined as the ear with the minimum maximal peak pressure difference (MPD) for each participant. The median MPD after swallowing was significantly lower in the ETD group than in the control group (0 daPa [IQR, 0–2] vs. 11 daPa [IQR, 6–17], *p* < 0.001), indicating markedly reduced pressure equalization ability in participants with ETD. The distribution of MPD values demonstrated clear separation between groups and is illustrated in Figure 2.

Consistent findings were observed in the per-ear analysis. The median MPD remained significantly lower in the ETD group compared with the control group (0 daPa [IQR, 0–3] vs. 15 daPa [IQR, 8–22.8], *p* < 0.001), supporting a distinct difference in objective ET function at the individual ear level.

Tympanometric findings also differed significantly between groups (*p* < 0.001). While all ears in the control group demonstrated Type A tympanograms (100.0%), tympanometric patterns in the ETD group were more heterogeneous, consisting of Type A (43.0%), Type B (38.0%), and Type C (19.0%). Notably, despite fulfilling the predefined diagnostic criteria for chronic ETD, 43.0% of ETD ears (34/79) remained classified as Type A. Tympanometric classification was recorded for descriptive purposes and was not incorporated into the diagnostic definition of ETD in this study.

### 3.3. Diagnostic Performance of Maximal Peak Pressure Difference (MPD) for Identifying Eustachian Tube Dysfunction

**Figure 3:**
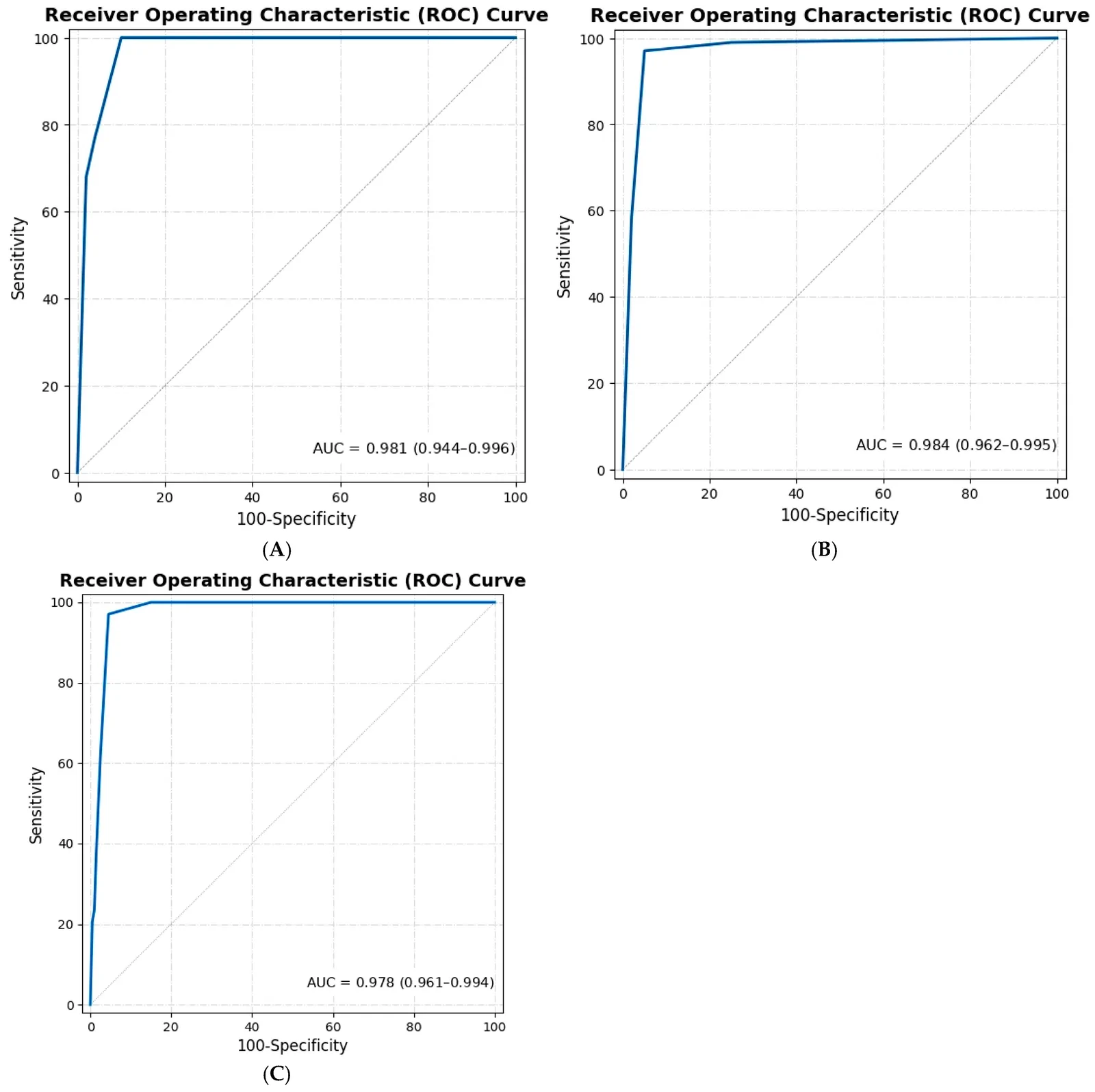
Receiver Operating Characteristic Curves of Maximal Peak Pressure Difference. Receiver operating characteristic (ROC) curve analyses demonstrating the diagnostic performance of MPD for discriminating chronic Eustachian tube dysfunction (ETD) from controls. The gray diagonal line represents the line of no discrimination (AUC = 0.5). (**A**) ROC curve for the per-person analysis, using the worst-performing ear for each participant, with an area under the curve (AUC) of 0.981 (95% CI, 0.944–0.996). (**B**) ROC curve for the per-ear analysis, with an AUC of 0.984 (95% CI, 0.962–0.995). In both analyses, the optimal cutoff value was identified as ≤4 daPa. (**C**) ROC curve for the Type A-only per-ear analysis, performed to evaluate the discriminatory performance of MPD among ears with normal resting tympanograms, with an AUC of 0.978 (95% CI, 0.961–0.994). In all analyses, the optimal cutoff value was identified as MPD ≤4 daPa.

Receiver operating characteristic (ROC) curve analysis was performed to determine the optimal MPD cutoff for identifying ETD. As shown in Figure 3 and summarized in Table 3, the predefined primary per-person analysis (*N* = 152) identified an MPD cutoff of ≤4 daPa, with a sensitivity of 100.0%, specificity of 91.0%, and an area under the curve (AUC) of 0.981 (95% CI, 0.944–0.996).

A supplementary exploratory per-ear analysis (*N* = 279) yielded the same optimal cutoff of ≤4 daPa, with a sensitivity of 97.5%, specificity of 95.5%, and an AUC of 0.984 (95% CI, 0.962–0.995). Because within-subject inter-ear correlation was not accounted for, these ear-level findings should be interpreted as supportive rather than primary evidence.

To further evaluate whether the discriminatory performance of MPD persisted after excluding abnormalities already detectable by conventional tympanometry, an additional ROC analysis was restricted to ears with Type A tympanograms (200 control ears and 34 ETD ears). MPD remained highly discriminative in this subgroup, with an AUC of 0.978 (95% CI, 0.961–0.994). The optimal cutoff remained ≤4 daPa, yielding a sensitivity of 97.1%, specificity of 95.5%, and Youden index of 0.926.

Overall, MPD demonstrated strong discriminatory ability in the predefined primary per-person analysis, with consistent supportive findings in both the supplementary per-ear and Type A-only analyses.

## 4. Discussion

The present study addresses a clinically relevant scenario frequently encountered in otologic practice, in which patients report symptoms suggestive of ETD, including persistent aural fullness, pressure disequilibrium, or impaired pressure equalization [27,28,29], despite demonstrating normal findings on conventional resting tympanometry (Type A tympanogram). Although tympanometry remains a widely used objective assessment of middle ear status, it primarily reflects middle ear pressure and tympanic membrane mobility under resting conditions and may not fully capture the dynamic opening behavior of the Eustachian tube during physiologic activities [1,6,30]. Consequently, subtle or functionally compensated ET abnormalities may remain undetected when assessment relies solely on static measurements.

This discrepancy may be particularly relevant in patients with mild, intermittent, or baro-challenge–related ETD [31], whose symptoms may become apparent primarily under conditions requiring active pressure regulation. In the present study, 43% of ETD ears (34 of 79 ears) demonstrated normal Type A tympanograms despite fulfilling both symptom-based and clinical diagnostic criteria for ETD [32]. This finding suggests that normal resting tympanometry does not necessarily exclude underlying functional impairment of the Eustachian tube, as conventional tympanometry primarily reflects static middle ear pressure and tympanic membrane mobility [1,10]. Subtle or compensated ETD may therefore remain undetected at rest and become apparent only when active pressure regulation is required. In this context, dynamic assessment of Eustachian tube function may provide complementary functional information beyond conventional resting tympanometry [3], supporting the potential role of pressure-swallow testing in symptomatic patients with otherwise unremarkable resting tympanometric findings.

Accordingly, the present study explored the pressure-swallow test implemented in the GSI TympStar Pro system as a complementary functional assessment for clinically suspected oETD. Rather than attempting to establish a universal physiological normative value, the ROC analysis was intended to identify a clinically applicable MPD cutoff for discriminating symptomatic oETD from healthy controls. Clinically diagnosed oETD, supported by the validated ETDQ-7 questionnaire and comprehensive otolaryngologic assessment, served as the clinical reference standard to reflect routine clinical practice.

Commercial implementation guidelines for the GSI TympStar Pro Eustachian Tube Function module have suggested that pressure shifts of approximately 15–20 daPa following swallowing generally represent adequate Eustachian tube opening and middle ear pressure equalization [33,34]. Accordingly, the reference MPD value initially suggested by the tympanometry manufacturer was approximately 15 daPa; however, this threshold has not been formally validated across different populations.

Previous studies have proposed different reference criteria for interpreting pressure-swallow testing and related inflation–deflation tympanometric assessments of Eustachian tube function. In the original description of Bluestone’s classical nine-step inflation/deflation test and subsequent early literature, no strict universal numerical cutoff for Eustachian tube dysfunction was proposed; instead, ET function was interpreted according to the degree of middle ear pressure equilibration achieved during swallowing following controlled inflation and deflation maneuvers [35,36]. A pressure change of approximately 10 mmH_2_O was generally considered indicative of functional tubal opening and pressure equalization. This conceptual reference was subsequently adopted in later studies, including those by McBride et al. [13,37] and Fernau et al. [14], for interpretation of nine-step inflation/deflation tympanometric test results.

Bae et al. (2022), in Revisiting the Diagnostic Performance of the Modified Nine-Step Test for Obstructive and Patulous Eustachian Tube Dysfunction, identified an ETTmd cutoff value of 13 daPa as the optimal diagnostic threshold for differentiating normal ears from ears with obstructive or patulous Eustachian tube dysfunction [15]. This cutoff provided the best overall diagnostic performance, with a specificity of 90% for both disease entities.

Ikeda et al. (2024), in the Japan Otological Society manual of Eustachian tube function tests [22], emphasized that objective ET function tests should be interpreted using representative waveform patterns together with established reference values. For the inflation–deflation test, a passive opening pressure (POP) of 150–550 daPa is considered the normal range, whereas markedly elevated or reduced values suggest obstructive or patulous Eustachian tube dysfunction, respectively.

Collectively, previous studies indicate that no universally accepted reference threshold exists for pressure-swallow–based assessment of Eustachian tube function, with reported values generally ranging from approximately 10 to 20 daPa. In the present prospective case–control study, ROC analysis identified an MPD cutoff of ≤4 daPa for discriminating clinically diagnosed symptomatic oETD from healthy controls, with strong discriminatory performance in the predefined primary per-person analysis. Although this cutoff is lower than previously reported values, it should be interpreted as a clinically derived discriminatory threshold rather than a universal physiological reference value.

Several methodological differences may explain this discrepancy. First, the manufacturer-recommended value of approximately 15–20 daPa appears to serve primarily as an operational reference rather than a population-validated diagnostic threshold. Second, previous studies differed substantially in study populations and case definitions. For example, Bae et al. derived a 13 daPa cutoff from a cohort in which oETD was defined by objective middle-ear abnormalities [15], including negative middle-ear pressure or tympanic membrane retraction, while ears without a measurable tympanometric peak, such as Type B tympanograms, were excluded. In contrast, our study focused on patients with persistent ETD-related symptoms, particularly aural fullness, regardless of resting tympanometric pattern. This broader clinical spectrum included patients with normal resting tympanograms as well as those with more apparent middle-ear abnormalities and may partly account for the lower ROC-derived cutoff.

Because Type B or Type C tympanograms were present in a substantial proportion of ETD ears, the overall discriminatory performance of MPD could partly reflect abnormalities already detectable by conventional tympanometry. To address this possibility, an additional ROC analysis was restricted to ears with Type A tympanograms. MPD remained highly discriminative in this subgroup (AUC, 0.978; 95% CI, 0.961–0.994), with the same optimal cutoff of ≤4 daPa. These findings support the potential complementary value of pressure-swallow testing in symptomatic patients with otherwise normal resting tympanometric findings. Nevertheless, the proposed cutoff was derived using the modified three-tympanogram pressure-swallow protocol implemented in the GSI TympStar Pro ETF–Intact module and should therefore not be directly generalized to the classical Bluestone nine-step test, other ET function testing systems, or different testing protocols.

Age-related confounding should also be considered because the ETD group was significantly older than the control group. In the exploratory age-stratified analysis, MPD remained significantly lower in the ETD group within each age stratum, suggesting that the observed difference was not solely attributable to the overall age imbalance. However, given the limited number of ETD participants in the younger strata, these findings should be regarded as supportive rather than definitive, and further validation using larger age-matched cohorts or multivariable age-adjusted models is warranted.

Overall, the markedly reduced MPD observed in symptomatic oETD is consistent with impaired middle-ear pressure equilibration during swallowing. When interpreted together with symptom assessment and routine clinical evaluation, pressure-swallow testing may therefore provide complementary objective information on dynamic ET function, particularly in patients with persistent symptoms despite normal resting tympanometry. Prospective multicenter studies are needed to determine the generalizability and clinical utility of this threshold across different populations and testing settings.

## 5. Limitations

This study has several limitations. First, it was conducted at a single tertiary referral center in Taiwan; therefore, the proposed MPD cutoff requires external validation in larger, multicenter studies and diverse populations before broader clinical application.

Second, the ETD group was significantly older than the healthy control group, and age may have confounded the observed differences in Eustachian tube function or MPD. Although exploratory age-stratified analysis showed that MPD remained significantly lower in the ETD group within each age stratum, the relatively small number of younger ETD participants limited robust multivariable adjustment. Future studies using larger age-matched cohorts or multivariable age-adjusted models are warranted.

Third, despite the use of predefined diagnostic criteria incorporating clinical assessment, otoscopic findings, tympanometric evaluation, and ETDQ-7 scores, the absence of a universally accepted objective gold standard for ETD diagnosis remains an inherent limitation [1,21].

Fourth, operators performing the pressure-swallow test were not formally blinded to group allocation or ETDQ-7 scores, which may have introduced assessment bias. However, MPD was automatically derived by the GSI TympStar Pro from device-recorded tympanometric peak pressure changes, thereby reducing subjective interpretation. Inter-rater and test–retest reliability were not assessed because repeated measurements were not routinely performed. Future prospective studies should incorporate blinded assessment and evaluate the reproducibility of MPD measurements.

Fifth, the proposed MPD cutoff was derived using the modified three-tympanogram pressure-swallow protocol implemented in the GSI TympStar Pro system and should therefore not be directly generalized to other ET function testing systems or protocols. In addition, the lowest MPD was selected for the primary per-person analysis to capture unilateral or asymmetric dysfunction; however, this approach may emphasize the most abnormal ear-level measurement, and side-specific symptom severity was not sufficiently standardized to support a robust symptomatic-ear analysis. Future studies should compare worst-ear, symptomatic-ear, and mean bilateral MPD approaches to determine the most appropriate patient-level summary measure.

Finally, because the supplementary per-ear analysis included bilateral observations from some participants without accounting for within-subject inter-ear correlation, these findings should be regarded as exploratory and supportive rather than primary evidence. Future ear-level analyses should account for clustering using appropriate statistical methods, such as generalized estimating equations or mixed-effects models.

## 6. Conclusions

The present single-center study suggests that dynamic pressure-swallow testing may provide complementary objective information for the functional assessment of patients with suspected obstructive Eustachian tube dysfunction, particularly when resting tympanometric findings are unremarkable. In this clinical setting, an MPD cutoff of ≤4 daPa identified using the GSI TympStar Pro pressure-swallow test may provide a clinically applicable discriminatory threshold for differentiating symptomatic obstructive ETD from healthy individuals in a Taiwanese population. Because this threshold was derived from a single-center cohort using a specific testing protocol, prospective multicenter studies are warranted to validate its generalizability before broader clinical application.

## Figures and Tables

**Table 1:** Participant Demographics and Baseline Characteristics.

Characteristic	Control Group	ETD Group	*p*-Value
A. Per-person characteristics	*n* = 100	*n* = 52	
Age, years			<0.001
Mean ± SD	36.0 ± 12.9	52.1 ± 16.5	
median (IQR)	32.0 (27.0–40.3)	54.5 (38.5–66.0)	
Range	21–79	20–78	
Sex, *n* (%)			0.305
Male	53 (53.0)	23 (44.2)	
Female	47 (47.0)	29 (55.8)	
B. Per-ear baseline characteristics	Ears, *n* = 200	Ears, *n* = 79	
Tympanogram type, *n* (%)			<0.001
Type A	200 (100.0)	34 (43.0)	
Type B	0 (0.0)	30 (38.0)	
Type C	0 (0.0)	15 (19.0)	

Note: The ETD group in the per-ear analysis included 79 symptomatic ears from 52 patients because some patients had bilateral involvement. In patients with unilateral symptoms, only the symptomatic ear was included; in patients with bilateral symptoms, both ears were included. Tympanogram type was recorded descriptively and was not used as an ear-level inclusion criterion. ETD: Eustachian Tube Dysfunction.

**Table 2:** Symptom Characteristics and Objective Pressure-Swallow Test Findings.

Outcome Measures	Control Group	ETD Group	*p*-Value
A. Per-person outcomes	*n* = 100	*n* = 52	
ETDQ-7 score			<0.001
Median	7.5	24.5	
IQR	7–9.8	17–32.8	
MPD, daPa			<0.001
Median	11	0	
IQR	6–17	0–2	
B. Per-ear outcomes	Ears, *n* = 200	Ears, *n* = 79	
MPD, daPa			<0.001
Median	15	0	
IQR	8–22.8	0–3	

Note: For the per-person analysis, the ear with the minimum maximal peak pressure difference (MPD) was selected for each participant to represent the worst-performing ear. ETD: Eustachian Tube Dysfunction; ETDQ-7: Eustachian Tube Dysfunction Questionnaire-7; MPD: Maximal Peak Pressure Difference.

**Table 3:** Diagnostic Performance of Maximal Peak Pressure Difference for Identifying Eustachian Tube Dysfunction.

Analysis Type	Sample Size, *N*	Sensitivity	Specificity	AUC (95% CI)	Youden Index
Per-person	152	100.0%	91.0%	0.981 (0.944–0.996)	0.91
Per-ear	279	97.5%	95.5%	0.984 (0.962–0.995)	0.93
Type A-only per-ear analysis	234	97.1%	95.5%	0.978 (0.961–0.994)	0.93

Note: The optimal cutoff value for maximal peak pressure difference was ≤4 daPa in all analyses. The per-person analysis was considered the primary analysis, whereas the per-ear and Type A-only per-ear analyses were supplementary exploratory ear-level evaluations. The Type A-only per-ear analysis included only ears with Type A tympanograms. Ear-level analyses did not account for within-subject inter-ear correlation and should therefore be interpreted with caution.

## Data Availability

The data presented in this study are available on reasonable request from the corresponding author due to privacy and ethical considerations regarding patient information.

## Supplementary Materials

The following supporting information can be downloaded at: https://www.mdpi.com/article/10.3390/diagnostics16172738/s1, Table S1: Age-Stratified Comparison of ETDQ-7 Scores and MPD Between the Control and ETD Groups.

## References

[1] Schilder A.G., Bhutta M.F., Butler C.C., Holy C., Levine L.H., Kvaerner K.J., Norman G., Pennings R.J., Poe D., Silvola J.T. (2015). Eustachian tube dysfunction: Consensus statement on definition, types, clinical presentation and diagnosis. Clin. Otolaryngol..

[2] Casale J., Shumway K.R., Hatcher J.D. (2018). Physiology, eustachian tube function. StatPearls.

[3] Smith M.E., Takwoingi Y., Deeks J., Alper C., Bance M.L., Bhutta M.F., Donnelly N., Poe D., Tysome J.R. (2018). Eustachian tube dysfunction: A diagnostic accuracy study and proposed diagnostic pathway. PLoS ONE.

[4] Swarts J.D., Alper C.M., Luntz M., Bluestone C.D., Doyle W.J., Ghadiali S.N., Poe D.S., Takahashi H., Tideholm B. (2013). Panel 2: Eustachian tube, middle ear, and mastoid—Anatomy, physiology, pathophysiology, and pathogenesis. Otolaryngol.—Head Neck Surg..

[5] Bluestone C.D. (1983). Eustachian tube function: Physiology, pathophysiology, and role of allergy in pathogenesis of otitis media. J. Allergy Clin. Immunol..

[6] Smith M.E., Tysome J.R. (2015). Tests of Eustachian tube function: A review. Clin. Otolaryngol..

[7] Sadé J., Ar A. (1997). Middle ear and auditory tube: Middle ear clearance, gas exchange, and pressure regulation. Otolaryngol.--Head Neck Surg..

[8] Bluestone C.D. (2005). Eustachian Tube: Structure, Function, Role in Otitis Media.

[9] Smith M.E., Bance M.L., Tysome J.R. (2019). Advances in Eustachian tube function testing. World J. Otorhinolaryngol. Head Neck Surg..

[10] Anastasiadou S., Bountzis P., Gkogkos D.E., Karkos P., Constantinidis J., Triaridis S., Psillas G. (2024). Eustachian Tube Dysfunction Diagnostic Pathway-What Is the Current State of the Art and How Relevant Is Chronic Nasal Disease?. J. Clin. Med..

[11] Alper C.M., Teixeira M.S., Mandel E.M., Swarts J.D. (2023). Dissecting eustachian tube dysfunction: From phenotypes to endotypes. PLoS ONE.

[12] Wang J.J., Jiang R.S., Weng C.H. (2024). Establishment of the Normative Value of Classical Bluestone’s Nine-Step Inflation/Deflation Tympanometric Eustachian Tube Function Test. Diagnostic.

[13] McBride T.P., Derkay C.S., Cunningham M.J., Doyle W.J. (1988). Evaluation of noninvasive eustachian tube function tests in normal adults. Laryngoscope.

[14] Fernau J.L., Hirsch B.E., Derkay C., Ramasastry S., Schaefer S.E. (1992). Hyperbaric oxygen therapy: Effect on middle ear and eustachian tube function. Laryngoscope.

[15] Bae S.H., Moon S., Jeong M., Moon I.S. (2022). Revisiting the Diagnostic Performance of the Modified Nine-Step Test for Obstructive and Patulous Eustachian Tube Dysfunction. Diagnostic.

[16] Uzun C., Adali M.K., Tas A., Koten M., Karasalihoglu A.R., Devren M. (2000). Use of the nine-step inflation/deflation test as a predictor of middle ear barotrauma in sports scuba divers. Br. J. Audiol..

[17] Schuchman G., Joachims H.Z. (1985). Tympanometric assessment of eustachian tube function of divers. Ear Hear..

[18] Tucci D.L., McCoul E.D., Rosenfeld R.M., Tunkel D.E., Batra P.S., Chandrasekhar S.S., Cordes S.R., Eshraghi A.A., Kaylie D., Lal D. (2019). Clinical Consensus Statement: Balloon Dilation of the Eustachian Tube. Otolaryngol. Head Neck Surg..

[19] Dave V., Ruparel M. (2019). Correlation of Eustachian Tube Dysfunction with Results of Tympanoplasty in Mucosal Type of Chronic Suppurative Otitis Media. Indian J. Otolaryngol. Head Neck Surg..

[20] Lin W.L., Chou Y.F., Sun C.H., Lin C.C., Hsu C.J., Wu H.P. (2020). Evaluation of thirty patients with eustachian tube dysfunction in Taiwan by questionnaire survey. J. Formos. Med. Assoc..

[21] McCoul E.D., Anand V.K., Christos P.J. (2012). Validating the clinical assessment of eustachian tube dysfunction: The Eustachian Tube Dysfunction Questionnaire (ETDQ-7). Laryngoscope.

[22] Ikeda R., Ohta S., Yoshioka S., Endo S., Lee K., Kikuchi T., Yoshida H., Inagaki A., Kaneko A., Kobayashi H. (2024). A manual of Eustachian tube function tests-illustration of representative test results obtained from healthy subjects and typical disorders with suggestion of the appropriate test method of choice. Auris Nasus Larynx.

[23] Nachar N. (2008). The Mann-Whitney U: A test for assessing whether two independent samples come from the same distribution. Tutor. Quant. Methods Psychol..

[24] McHugh M.L. (2013). The chi-square test of independence. Biochem. Medica.

[25] Mandrekar J.N. (2010). Receiver operating characteristic curve in diagnostic test assessment. J. Thorac. Oncol..

[26] Youden W.J. (1950). Index for rating diagnostic tests. Cancer.

[27] Shan A., Ward B.K., Goman A.M., Betz J.F., Reed N.S., Poe D.S., Nieman C.L. (2019). Prevalence of Eustachian Tube Dysfunction in Adults in the United States. JAMA Otolaryngol. Head Neck Surg..

[28] Altalhi W.A., Alsulaimani A.I., Alhossaini Z.A., Alharthi R.M., Almalki Z.A., Altalhi W.A., Alswat S.H., Alnefaie G.O. (2022). Prevalence of and Factors Associated With Eustachian Tube Dysfunction Among the Public in Taif, Saudi Arabia. Cureus.

[29] Tangbumrungtham N., Patel V.S., Thamboo A., Patel Z.M., Nayak J.V., Ma Y., Choby G., Hwang P.H. (2018). The prevalence of Eustachian tube dysfunction symptoms in patients with chronic rhinosinusitis. Int. Forum Allergy Rhinol..

[30] Todd N.W. (2000). There are no accurate tests for eustachian tube function. Arch. Otolaryngol. Head Neck Surg..

[31] Hamrang-Yousefi S., Ng J., Andaloro C. (2023). Eustachian tube dysfunction. StatPearls [Internet].

[32] Andresen N.S., Sharon J.D., Nieman C.L., Seal S.M., Ward B.K. (2021). Predictive value of the Eustachian Tube Dysfunction Questionnaire-7 for identifying obstructive Eustachian tube dysfunction: A systematic review. Laryngoscope Investig. Otolaryngol..

[33] Grason-Stadler I. Pressure-Swallow Test. Grason-Stadler, Inc. https://grason-stadler.com/education/guides/eustachian-tube-function.

[34] Laura Prigge A. How Do I Perform a Eustachian Tube Function Test?. https://www.audiologyonline.com/ask-the-experts/do-i-perform-eustachian-tube-24752.

[35] Bluestone C.D. (2004). Studies in otitis media: Children’s Hospital of Pittsburgh–University of Pittsburgh progress report—2004. Laryngoscope.

[36] Jerger J. (1975). Handbook of Clinical Impedance Audiometry.

[37] McBride T.P., Doyle W.J., Hayden F.G., Gwaltney J.M. (1989). Alterations of the eustachian tube, middle ear, and nose in rhinovirus infection. Arch. Otolaryngol. Head Neck Surg..

