# Supplementary material for: Functional Assessment Beyond Type A Tympanograms: Pressure-Swallow Testing for Chronic Eustachian Tube Dysfunction in a Taiwanese Cohort
Source: Diagnostics (Basel). 2026 Aug 26;16(17):2738. doi: 10.3390/diagnostics16172738 (PMC13564953; doi:10.3390/diagnostics16172738)
Supplement: Supplementary file 1 [file diagnostics-16-02738-s001.zip › diagnostics-4436396-supplementary.pdf]

Table S1. Age-Stratified Comparison of ETDQ-7 Scores and MPD Between the Control and ETD Groups.

Age Stratum	Variable	Control Group, Median (Q1–Q3)	ETD Group, Median (Q1–Q3)	*p*-Value
Age 20–30 years (*n* = 43 vs. 8)	Age, years	27.0 (24.0–28.0)	27.0 (22.3–28.5)	0.685
	ETDQ-7 score	7.0 (7.0–10.0)	24.0 (16.3–32.8)	<0.001
	MPD, daPa	13.0 (9.0–20.0)	1.5 (0.0–3.8)	<0.001
Age 31–46 years (*n* = 41 vs. 10)	Age, years	35.0 (32.0–40.0)	38.0 (33.8–44.3)	0.238
	ETDQ-7 score	8.0 (7.0–9.5)	30.0 (17.0–32.5)	<0.001
	MPD, daPa	8.0 (5.0–14.0)	0.0 (0.0–2.3)	<0.001
Age >46 years (*n* = 16 vs. 34)	Age, years	60.0 (49.5–70.3)	63.0 (54.8–69.0)	0.532
	ETDQ-7 score	7.0 (7.0–9.8)	23.5 (18.5–33.0)	<0.001
	MPD, daPa	11.5 (5.3–22.5)	0.0 (0.0–2.0)	<0.001

Note: Data are presented as median (Q1–Q3). Group comparisons were performed using the Mann–Whitney U test. ETD, Eustachian tube dysfunction; ETDQ-7, Eustachian Tube Dysfunction Questionnaire-7; MPD, maximal peak pressure difference.
